# Brain–Computer Interfaces, Completely Locked-In State in Neurodegenerative Diseases, and End-of-Life Decisions

**DOI:** 10.1007/s11673-023-10256-5

**Published:** 2023-07-19

**Authors:** Christopher Poppe, Bernice S. Elger

**Affiliations:** 1 Institute for Biomedical Ethics, University of Basel, Bernoullistr. 28, 4056 Basel, Switzerland; 2 Center for Legal Medicine of Geneva and Lausanne, Geneva, Switzerland

**Keywords:** Brain, computer interfaces, Assisted suicide, Withdrawing treatment, End of life, ALS, Locked-in state

## Abstract

In the future, policies surrounding end-of-life decisions will be faced with the question of whether competent people in a completely locked-in state should be enabled to make end-of-life decisions via brain-computer interfaces (BCI). This article raises ethical issues with acting through BCIs in the context of these decisions, specifically self-administration requirements within assisted suicide policies. We argue that enabling patients to end their life even once they have entered completely locked-in state might, paradoxically, prolong and uphold their quality of life.

## Background

In the last decade, brain–computer interfaces (BCI) have become practically useful for communication and movement of people with brain injury or neurodegenerative diseases (Holz, et al. 2015; Kübler 2020). Acting and communicating through BCIs has turned from fiction to reality. The role of communication and acting via BCI in end-of-life decision-making unwraps several ethical issues, most vitally agency, autonomy, and responsibility for actions via BCIs. In a recent article, Rainey and colleagues (Rainey, Maslen, and Savulescu 2020) outlined moral responsibility for action through BCIs, understanding them as “willed bodily movement.” Acting through BCIs is particularly important for people who cannot move otherwise, a state referred to as locked-in state (Bernat 2020). Generally, locked-in state has traditionally been separated into incomplete locked-in, classical locked-in (LIS), and total or completely locked-in (CLIS) state (Bernat 2020). Incomplete LIS includes further muscle movement other than gaze and eyelid while the clinical syndrome of classical LIS consists of “quadriplegia, lower cranial nerve paralysis, and mutism with preservation of consciousness, vertical gaze, and upper eyelid movement” (Plum and Posner 1966; Smith and Delargy 2005). LIS is most often caused by stroke and disorder of the brain stem, especially the pons (Bernat 2020). However, neurodegenerative diseases, for example amyotrophic lateral sclerosis (ALS), leading to paralysis and quadriplegia have been included in the definition of LIS (Bernat 2020). In the absence of an earlier death, CLIS occurs in the final stage of ALS and people with ALS in CLIS lack all voluntary muscle movement even eye movement (Hayashi and Kato 1989). In these cases, actions via BCI can prove to be the only means to exercise autonomy during end-of-life decision-making.

However, people in CLIS are not only metaphorically locked-in but they are also “through social exclusion, stigmatization, and frequently being underestimated in their abilities, […] locked out,” as Johansson and colleagues have argued (Johansson, Soekadar, and Clausen 2017, 555). Underestimating the ability of people in CLIS to make decisions only due to lack of motor control while having decisional capacity would be discriminating against these patients (Glannon 2016). Thus, decision-making with actions and communication through BCI at the end-of-life is at the centre of this article. We highlight multiple ethical and practical issues potentially restricting the use of BCIs for autonomous decision-making of people in CLIS at the end of life, especially with assisted suicide policies based on the self-administration. However, we argue that there are cases where these restrictions should not apply and hence, some people in CLIS should be allowed to end their own life, either through withdrawal of treatment or assisted suicide controlling the lethal substance intake via BCI.

### Acting Through Brain–Computer Interfaces

For the discussion of use of BCIs at the end of life, it is necessary to provide a short overview of types and functions of BCIs (for an extensive introduction, see Wolpaw and Wolpaw 2012). Generally, BCIs establish a connection between the human brain and a technological device based on electrical activity. One can distinguish BCIs by the direction of information flow from and to the brain. For instance, any BCI processes input data from the brain to output data in a communicative device (e.g., a spelling device) or neuroprosthetic assistive device (e.g., wheelchair, robot hand) (Linse, et al. 2018), without providing feedback to the brain. Other BCIs such as Deep Brain Stimulators (DBS) provide electrical stimulation to brain tissue. More recent BCIs bridge both directions and not only process input data but also stimulate the brain according to certain input neural activity with or without the control of the user (e.g., in epilepsy, Kellmeyer, et al. 2016).

BCIs can use different neural data from invasive and non-invasive sources, for example from functional magnet resonance imaging (fMRI), non-invasive electroencephalography (EEG), invasive electrocorticography (ECoG), or functional near-infrared spectroscopy (fNIRS). In most BCIs, electrical brain activity sampled via EEG or ECoG is amplified, digitally processed, and computed. BCIs are therefore necessarily limited by the kind of data they process and its temporal and spatial accuracy.

BCIs can be distinguished by the way this data is computed by the BCI. Passive BCIs do not need any user action and passively classify neural activity, leading to potential problems with the user’s agency, especially within closed-loop systems providing neurostimulation if people feel “as if they are no longer fully in control of their own actions” (Schönau, et al. 2021, 181; see also Kellmeyer, et al. 2016). In active BCIs, neural activity corresponding to a mental task, e.g., imagination of movement, is needed to enable output of the BCI as action or communication (Steinert, et al. 2019). In reactive BCIs, it is the neural activity reacting to a presented stimulus that is classified as relevant by the BCI. Among these reactive devices, the event-related potential based P300-spelling device has been studied and used extensively, however its classification accuracy is contested (Marchetti and Priftis 2014). As most BCIs use EEG, we will focus on BCIs applications in this tradition and our theoretical arguments are sketched out with a P300-speller in mind.

Brain–computer interfaces require algorithmic processing and pattern recognition for classification of brain signals based on probabilistic processes and machine learning (Mainsah, et al. 2015). Hence, there is an intrinsic uncertainty in decision-making via BCI and similar ethical issues arise as with the use of algorithms more widely (Wolkenstein, Jox, and Friedrich 2018). For accuracy, most algorithms and users of BCIs must be trained over time (Lotte, et al. 2018). This necessarily complicates BCI use in cases were the need for the use of the BCIs appears suddenly (e.g., after traumatic brain injury or stroke). However, in cases where CLIS is foreseeable, the individual training of algorithms is possible and safeguarded by other means of communication available in earlier stages of progressive diseases. Uncertainty in decision-making is necessarily higher if communication via BCI relies on simple yes/no answers, rather than complete sentences (Peterson et al. 2013).

## Ending One’s Life Through Brain–Computer Interfaces

### Withdrawing Treatment or Assisted Suicide

People in CLIS are ventilator-dependent and in need of artificial feeding. Hence, if they have decision-making capacity, they do not need to commit AS, as withdrawal of treatment offers them a viable way of ending their life.^1^ Unlike assisted suicide, the latter is legal in the vast majority of countries. But also, beyond legal considerations, switching off life supporting machines while being supported by supportive care might seem more reasonable than administering pentobarbital through a gastric catheter. Still, for the purpose of this article, we focus on assisted suicide via BCI since we believe that our arguments concerning assisted suicide could be similarly applied to withdrawal of treatment. The most notable difference in assisted suicide and withdrawal of treatment is that assisted suicide requires a wilful action (the person’s physical control of the pharmakon), while withdrawal of treatment solely needs the communication of treatment preference to the attending physicians (i.e., for the ending of treatment). In this way, assisted suicide has more extensive normative requirements. It is central to our argument that if these requirements can be met for assisted suicide, they can also be met for withdrawal of treatment.

In addition, some patients in CLIS might also have reasons to prefer assisted suicide, e.g., because it gives them better control about the time and mode of their death. Similar to other patients, patients in CLIS should have the choice between assisted suicide and withdrawal of treatment, where assisted suicide is legal. This choice however is subject to having decision-making capacity and, in assisted suicide, having physical control.

### Decision-Making at the End-of-Life: The Example of Self-Administration in Assisted Suicide

Self-administration by the person requesting assisted suicide is a fundamental requirement in most countries where assisted suicide is legal, including in Switzerland. Assisted suicide is legal in Switzerland if the assistance is not provided because of selfish motives according to the Swiss Penal Code, Article 115. Access to AS requires decision-making capacity (henceforth, capacity) and physical control over the administration of the lethal drug. Physical control can go as far as having a mechanical device linked to muscle activity opening a gastric catheter containing pentobarbital (Bosshard et al. 2003). This device renders it possible to “provide an easy-to-handle remote control which they can activate with a small movement (e.g. a finger, toe or jaw) to start the attached pump”^2^. In CLIS, this movement is impossible and hence this small movement could only be replaced by action through a BCI.

Internationally, self-administration has been introduced as a safeguard in many countries that allow for assisted suicide, for example it has been a core component in policies on assisted dying since the 1994 Death with Dignity act in Oregon and several other states in the United States which have instated AS policies since then (Thyden 2017). In the Australian state of Victoria, self-administration is preferred, however, if “the patient is physically incapable of self-administering or digesting the voluntary assisted dying medication then the coordinating medical practitioner may assist the patient to die by administering the medication” (Victoria State Government 2021). Several other countries have instated both self-administration and practitioner-administration in policies on assisted suicide (British Medical Association 2021).

In strict self-administration policies, both criteria, capacity and control, serve to safeguard autonomy in these end-of life decisions. However, control is in some cases of CLIS an unsurmountable obstacle; more specifically for people with CLIS in the context of amyotrophic lateral sclerosis and other neurodegenerative diseases. Similar problems exist in Huntington’s disease. From the perspective of disability rights, Alicia Ouellette (2017, 5) has argued that

The requirement of self-administration poses particular problems for Huntington’s sufferers. Huntington’s disease is a progressive, neurodegenerative disorder that is inevitably fatal. Marked by involuntary movements, swallowing disorders, the inability to speak, and cognitive impairments, Huntington’s patients may lose the ability to self-ingest prescription medicine before they are eligible for PAD [physician-assisted dying], by being deemed within six months of death, or by completing the multistep process of approvals. The self-ingestion requirement becomes an impenetrable barrier for those seeking PAD, leading families of individuals with Huntington’s to argue in court briefs that the so-called protective procedures discriminate based on disability status.

In ALS however, the progressive nature necessitates invasive ventilation for long-term survival (Hayashi and Oppenheimer 2003) and a thorough evaluation of capacity.

### Capacity as a Gatekeeper for BCI-Assisted Suicide—The Case of ALS

Decision-making capacity and its different components (e.g., understanding or reasoning) can be established by the treating physician through unstructured or structured interviews (MacCAT-T; Grisso, et al. 1997) but remain reliant on a clinical judgement by the physician (Hermann et al. 2020). Hence, in most cases of CLIS from traumatic brain injury leading to minimal conscious states (MCS), capacity is difficult to establish because of the lack of adequate and reliable communication. For example, Cabral and Illes (2017) argue that communication via fMRI imaging fails to establish capacity in patients with traumatic brain injuries in general. However, as pointed out earlier, not all patients in CLIS have traumatic brain injuries and only a very small number of BCIs are based on fMRI. Nonetheless, the general point stands, that in certain cases of CLIS where communication via BCI is unreliable, any evaluation of decision-making capacity will be difficult.

Typically, when it is impossible to assess capacity, surrogates will decide about possible end-of-life options. In cases of severe brain injury where capacity is unclear, Joseph Fins has argued for a more nuanced approach to assess capacity in minimally conscious states (MCS), encompassing both surrogate decision-making and self-determination via neuroprosthetics. Fins calls this the “mosaic approach to decision-making, which seeks to achieve a proportionate and prudent balance between unbridled self-determination and conventional surrogate representation” (Fins 2018, 164).

This mosaic approach to decision-making and other approaches to decision-making of people in MCS have been discussed widely (Fins 2018; Peterson 2019; Fins 2019). Walter Glannon has argued for a “a weaker version of informed consent and decisional capacity” for “minimally conscious patients with a higher level of awareness and cognitive function who can clearly express their preferences about life-sustaining care through BCI-mediated binary responses” (Glannon 2016, 5).

However, there are more clear-cut cases where capacity could be preserved in CLIS. This is the case of amyotrophic lateral sclerosis. ALS is generally recognized as a terminal neurodegenerative disease of the motor neurons with progressive muscle weakness and atrophy requiring invasive mechanical ventilation and leading to first LIS, then CLIS. While in LIS, eye movement is preserved, enabling communication by eye movement. However, barring an earlier death, some patients suffering from ALS unfortunately enter CLIS (Murguialday, et al. 2011). While population-based studies show that almost half of people with ALS remain cognitively unimpaired (Phukan, et al. 2012), some even so in CLIS (Fuchino et al. 2008), cognitive impairment including frontotemporal dementia is nowadays recognized as a hallmark of ALS (Giordana, et al. 2011; Pender, Pinto-Grau, and Hardiman 2020) and worsens with the progression of the disease, especially when there is bulbar impairment (Chiò, et al. 2019). Full-blown dementia with major frontotemporal impairment can be diagnosed in 14 per cent of people with ALS (Pender, Pinto-Grau, and Hardiman 2020) encompassing symptoms such as “personality change, irritability, obsessions, poor insight, and pervasive deficits on frontal executive tests” (Phukan, Pender, and Hardiman 2007, 994). Given the heterogenous nature of cognitive impairment in ALS, it is important to evaluate capacity individually in each case. As some forms of BCI communication are sufficiently reliable (e.g., P300-speller) and have been used for cognitive testing (Lulé, et al. 2018), this can enable capacity evaluation.

If reliable communication can be established through a P300-speller BCI, this would permit to distinguish between absence and presence of capacity for different types of decision-making, including end-of-life decisions. However, if we only think of communication within narrow constraints on the complexity via BCI, for example the binary responses envisioned by Glannon (2016), there will probably be a number of cases where it is impossible to establish whether a person has capacity to make an informed decision or not. Using a BCI spelling device, capacity can be established in the same way it would in non-locked in people showing cognitive impairment. The process of capacity evaluation, however, would be more time consuming.

### Diachronic Autonomy of Individuals With ALS in CLIS

In contrast to the rapid nature of traumatic brain injury, in neurodegenerative diseases decision-making has an extended temporal dimension. In contrast to other cases of CLIS, the progressive nature of ALS enables anticipating CLIS from diagnosis on. People with ALS could therefore prepare end-of-life decisions in advance, as with the withdrawal of treatment (Moss et al. 1996). However, an advance directive requiring assisted suicide is not sufficient due to the need to self-administer the drug. One could ask whether, from an ethical standpoint, on the one hand the presence of an advance directive on assisted suicide in these ALS patients in CLIS would permit to accept some uncertainty in capacity evaluations via BCI. On the other hand, the advance directive could also only be procedural, directing the terms necessary to guide capacity evaluation and assisted suicide during CLIS.

This means that if people with ALS have a very stable wish to decide their own fate if they ever enter CLIS, a lower standard of communication could be deemed acceptable because they just reiterate a previously stable wish. Indeed, ALS might be the only case where the reliability of communication and the training of algorithms on BCI data is feasible because individuals can still communicate well before CLIS. Furthermore, training of the algorithm is also possible as long as people with ALS in LIS have not advanced to CLIS with the convergence of eye-tracking communication and BCI communication. This might give more certainty and credibility to the evaluation of capacity and communication of choice via BCI later.

### Physical Control and Self-Administration via BCI

Even if capacity could be established in some cases of ALS, physical control of the intake of the lethal substance must also be established, as this remains a major safeguard for autonomy in self-administered assisted suicide. Prima facie, there seems to be a plausible case to treat BCI control and ordinary muscular control the same because legally, responsibility for misconduct via BCI is attributed to the person using the BCI (Bublitz, et al. 2018). Bringing about harm or damage to other persons or objects with the use of BCI does not exempt the person who used it from legal ramifications. Physical control, tort liability, and criminal responsibility are innately linked and that, if users of BCIs (e.g. patients in CLIS) are liable for actions committed against others (Thompson 2019), they should be able to be responsible for acts against themselves (e.g. assisted suicide), too. Rainey and colleagues (Rainey, Maslen, and Savulescu 2020) have argued that while foreseeability and issues of control distinguish BCI actions to some extent from conventional actions, in cases where “there is a clear reason for the use of a BCI technology for assistance or owing to disability, it appears easy to assimilate this kind of action” (56). For the context of control via BCI in CLIS, the reasons for its use are clear: there is no other option for enacting one’s end of life. On this view, end-of-life decisions enacted via BCI are the only option of patients in CLIS.

## Discussion

We have argued that in some specific cases of CLIS, namely in patients developing CLIS in the context of ALS and wishing to end their lives, assisted suicide via BCI could be an option if withdrawal of treatment is not preferred. This sensitive topic demands several caveats. The first is that enabling the option of assisted suicide and withdrawal of treatment in CLIS, does not mean that life is not worth living in CLIS. Indeed, there are reports of a good quality of life for people with ALS in LIS who were able to communicate (Holz, et al. 2015; Kuzma-Kozakiewicz, et al. 2019; Lulé, et al. 2009). Generally, this quality of life and a sense of control might be improved by the use of BCIs (Gilbert, et al. 2019). Moreover, research shows that quality of life in LIS is sometimes underestimated by next of kin (Aust, et al. 2022).

The option for BCI-assisted suicide should therefore rather be understood as part of several strategies to increase autonomy and enable choice concerning a good life in CLIS. With this option for BCI-assisted suicide, among others, it might indeed be reasonable that more people with ALS enter CLIS—and do not feel forced to die before their quality-of-life declines to a point where they do not wish to live any longer. Indeed, they would not be forced to decide for assisted suicide or against invasive ventilation before they develop LIS, especially if quality of life may still be maintained there. For example, the dilemma of early assisted suicide is retold in a recent article by Andrea Kübler (2020, 174)

I remember vividly an ALS patient in LIS who was trained with SCP [slow cortical potentials] during the research for my doctoral thesis. He still enjoyed life, but made very clear that he desired assisted suicide before entirely losing motor capacity. This was also the case for another ALS patient just recently encountered.

We can only speculate whether these patients would have decided for BCI-assisted suicide or withdrawal of treatment at a later time (e.g., during LIS or CLIS) when these end-of-life options would have existed through a BCI. These examples also show the possibility to safeguard autonomous decision-making because the patients had been trained in using the BCI before, thereby providing a safeguard against ill-controlled use of the BCI. Generally, it has been postulated that quality of life of people in CLIS might be even improved due to the availability of end-of-life options as the choice provides some control over the current situation even if they do not wish to die (Colburn 2020).

A second caveat relates to the state of BCI research. While there have been reports of established independent use of BCIs in ALS (Wolpaw, et al. 2018), the research field has also had a major research ethics scandal with the alleged research misconduct committed by Birbaumer and colleagues on exactly the topic of communication with paralyzed ALS patients (Spüler 2019; Chaudhary, et al. 2017). Not only does this leave open the question of effectiveness of establishing BCI communication in these patients, but it will also most likely have undermined trustworthiness for intricate decisions via BCI at the end-of-life for patients and their families. Robust and replicable research procedures (as by the same researchers, Chaudhary et al. 2022) would therefore need to establish the viability of BCI communication before it could be applied in the context of end-of-life decision-making.

## Conclusion and Future Research

End-of-life decision-making, especially with regard to assisted suicide is riddled with complex ethical issues even without the involvement of BCIs. Considering all the caveats, implications for current policies on the use of BCIs and the requirement of self-administration can be sketched out.

The use of BCIs at the end of life needs to be planned early in the disease trajectory to enable diachronic autonomy in decision-making. Hence, when available, BCIs should be recommended and discussed by the treating multidisciplinary team in the context of neurodegenerative diseases, however, due to the novelty of the field it is unclear whether certain patient groups might profit more from having an BCI implanted early than others.

Furthermore, advance care planning should encompass preferred decisions that users want to perform with the BCI, as well as general preferences for end-of-life treatment.

One further conclusion could be to demand for self-administration to be scraped as a requirement for assisted suicide. Indeed, as outlined above, in some jurisdictions policies allow for practitioner administration (euthanasia) in cases where the person is unable to self-administer. Hence, due to the difficulties with acting through BCIs, self-administration could be abandoned and BCIs would only be used to request practitioner-administration of a lethal drug. While there are several reasons why BCI-assisted suicide might be preferable to euthanasia (e.g., feeling in control over one’s death), there is a lack of research on end-of-life preferences in CLIS pointing to a need for further research.

BCI are something entirely new “under the sun” (Wolpaw and Wolpaw 2012). As such, they bring forward the possibility of entirely new forms of action. When neural activity can be algorithmically transformed into communication, this communication could be used to cause action. Consider the similar everyday case without the use of a BCI: a user types a password into a computer and the result is the unlocking of the computer. Here, the neural activity of memory and communication of its content cause an action. It is not unreasonable to think that in decision-making with BCIs something similar could be applied. Entering a password, or a longer sentence, with a spelling device could prompt a computer to open the drip of a lethal drug, and hence act as self-administration.^3^ These normative questions of action and communicative agency are beyond the scope of this paper and should be subject to further philosophical and empirical research.

In conclusion, we have argued that BCIs can be of use for end-of-life decision-making in CLIS and that acting via BCIs should have the same normative status as muscular self-administration in the context of assisted suicide. Finally, enabling patients to end their life even once they have entered CLIS might, paradoxically, prolong and uphold their quality of life.

## Data Availability

Not applicable.

## Footnotes

1 In some cases of neurostimulating BCIs, even deactivating the BCI might lead to the death of the user and offer another way of ending one’s life. For example, while deep brain stimulation for Parkinson disease improves quality of life, it also prolongs the disease trajectory leading to novel ethical questions regarding deactivation of DBS in these cases (Gilbert and Lancelot, 2021; Sankary et al. 2020). We thank one of the anonymous reviewers for this suggestion.

2 Dignitas. 2014. How Dignitas works [brochure]. http://www.dignitas.ch/images/stories/pdf/so-funktioniert-dignitas-e.pdf. Accessed May 15, 2020.

3 This kind of device is not new. The so-called Deliverance Machine invented by Dr Philip Nitschke for assisted suicide in the 1990s basically performs the same process with several prompts of a “Yes” button (ILoveUselessKnowledge. 2014. The “Deliverance Machine” , invented by Philip Nitschke. *Exit International*, September 14. https://www.exitinternational.net/the-deliverance-machine-invented-by-philip-nitschke/. Accessed October 10, 2021. As evident from this article, a button would not suffice in the context of CLIS due to the uncertainty of BCI-action.
